# Tau and neurofilament light‐chain as fluid biomarkers in spinocerebellar ataxia type 3

**DOI:** 10.1111/ene.15373

**Published:** 2022-05-26

**Authors:** Hector Garcia‐Moreno, Mercedes Prudencio, Gilbert Thomas‐Black, Nita Solanky, Karen R. Jansen‐West, Rana Hanna AL‐Shaikh, Amanda Heslegrave, Henrik Zetterberg, Magda M. Santana, Luis Pereira de Almeida, Ana Vasconcelos‐Ferreira, Cristina Januário, Jon Infante, Jennifer Faber, Thomas Klockgether, Kathrin Reetz, Mafalda Raposo, Ana F. Ferreira, Manuela Lima, Ludger Schöls, Matthis Synofzik, Jeannette Hübener‐Schmid, Andreas Puschmann, Sorina Gorcenco, Zbigniew K. Wszolek, Leonard Petrucelli, Paola Giunti

**Affiliations:** ^1^ Ataxia Centre Department of Clinical and Movement Neurosciences UCL Queen Square Institute of Neurology University College London London UK; ^2^ Department of Neurogenetics National Hospital for Neurology and Neurosurgery University College London Hospitals NHS Foundation Trust London UK; ^3^ Department of Neuroscience Mayo Clinic Jacksonville Florida USA; ^4^ 156400 Neuroscience Graduate Program Mayo Clinic Graduate School of Biomedical Sciences Jacksonville Florida USA; ^5^ Department of Neurology Mayo Clinic Jacksonville Florida USA; ^6^ 61554 Department of Neurodegenerative Disease UCL Queen Square Institute of Neurology London UK; ^7^ UK Dementia Research Institute at UCL London UK; ^8^ Department of Psychiatry and Neurochemistry Institute of Neuroscience and Physiology the Sahlgrenska Academy at the University of Gothenburg Mölndal Sweden; ^9^ Clinical Neurochemistry Laboratory Sahlgrenska University Hospital Mölndal Sweden; ^10^ 37829 Center for Neuroscience and Cell Biology University of Coimbra Coimbra Portugal; ^11^ Coimbra University Hospital Centre Coimbra University Coimbra Portugal; ^12^ Neurology Service University Hospital Marqués de Valdecilla‐IDIVAL University of Cantabria Centro de Investigación en Red de Enfermedades Neurodegenerativas (CIBERNED) Santander Spain; ^13^ 39062 Department of Neurology University Hospital Bonn Bonn Germany; ^14^ German Center for Neurodegenerative Diseases (DZNE) Bonn Germany; ^15^ Department of Neurology RWTH Aachen University Aachen Germany; ^16^ JARA‐BRAIN Institute Molecular Neuroscience and Neuroimaging Forschungszentrum Jülich RWTH Aachen University Aachen Germany; ^17^ Faculdade de Ciências e Tecnologia Universidade dos Açores Ponta Delgada Portugal; ^18^ Instituto de Biologia Molecular e Celular (IBMC) Instituto de Investigação e Inovação em Saúde (i3S) Universidade do Porto Porto Portugal; ^19^ Department for Neurodegenerative Diseases Hertie‐Institute for Clinical Brain Research and Center for Neurology University of Tübingen Tübingen Germany; ^20^ German Center for Neurodegenerative Diseases (DZNE) Tübingen Germany; ^21^ Institute of Medical Genetics and Applied Genomics University of Tübingen Tübingen Germany; ^22^ Lund University, Skåne University Hospital Clinical Sciences, Neurology Lund Sweden

**Keywords:** biomarkers, cerebellum, neurofilaments, spinocerebellar ataxias, tau

## Abstract

**Background and purpose:**

Clinical trials in spinocerebellar ataxia type 3 (SCA3) will require biomarkers for use as outcome measures.

**Methods:**

To evaluate total tau (t‐tau), glial fibrillary acidic protein (GFAP), ubiquitin carboxy‐terminal hydrolase L1 (UCHL1) and neurofilament light‐chain (NfL) as fluid biomarkers in SCA3, *ATXN3* mutation carriers (*n* = 143) and controls (*n* = 172) were clinically assessed, and the plasma concentrations of the four proteins were analysed on the Simoa HD‐1 platform. Eleven *ATXN3* mutation carrier cerebrospinal fluid samples were analysed for t‐tau and phosphorylated tau (p‐tau^181^). A transgenic SCA3 mouse model (MJDTg) was used to measure cerebellar t‐tau levels.

**Results:**

Plasma t‐tau levels were higher in mutation carriers below the age of 50 compared to controls, and the Inventory of Non‐Ataxia Signs was associated with t‐tau in ataxic patients (*p *= 0.004). Pre‐ataxic carriers showed higher cerebrospinal fluid t‐tau and p‐tau^181^ concentrations compared to ataxic patients (*p *= 0.025 and *p *= 0.014, respectively). Cerebellar t‐tau was elevated in MJDTg mice compared to wild‐type (*p *= 0.033) only in the early stages of the disease. GFAP and UCHL1 did not show higher levels in mutation carriers compared to controls. Plasma NfL concentrations were higher in mutation carriers compared to controls, and differences were greater for younger carriers. The Scale for the Assessment and Rating of Ataxia was the strongest predictor of NfL in ataxic patients (*p *< 0.001).

**Conclusion:**

Our results suggest that tau might be a marker of early disease stages in SCA3. NfL can discriminate mutation carriers from controls and is associated with different clinical variables. Longitudinal studies are required to confirm their potential role as biomarkers in clinical trials.

## Graphical abstract

Plasma total tau (t‐tau) is elevated in young *ATXN3* mutation carriers compared to controls, and cerebrospinal fluid t‐tau and phosphorylated tau^181^ may be higher in pre‐ataxic subjects compared to ataxic patients. In addition, cerebellar t‐tau levels seem to be increased in early stages of the disease in a transgenic spinocerebellar ataxia type 3/Machado–Joseph disease (SCA3/MJD) mouse model (MJDTg). Therefore, tau could be a marker of early stages in SCA3/MJD. Plasma neurofilament light‐chain (NfL) concentrations are higher in *ATXN3* mutation carriers compared to controls, and such difference is more marked for younger subjects. Plasma NfL concentrations were associated with clinical variables and are a good candidate as a fluid biomarker in SCA3/MJD.

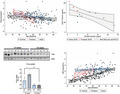

## INTRODUCTION

Spinocerebellar ataxia type 3/Machado–Joseph disease (SCA3/MJD) is the most common autosomal dominant cerebellar ataxia worldwide and is caused by a CAG repeat expansion in the *ATXN3* gene, which encodes ataxin‐3 [1, 2]. Currently, there is no curative treatment for this condition and trials for compounds targeting the mutant *ATXN3* allele will require disease markers for use as outcome measures [3, 4, 5]. Tau, neurofilament light‐chain (NfL), glial fibrillary acidic protein (GFAP) or ubiquitin carboxy‐terminal hydrolase L1 (UCHL1) could address this unmet need for biofluid markers, as they can mirror neuronal injury or underlying pathological processes in the central nervous system [6].

The microtubule‐associated protein tau (encoded by the *MAPT* gene) has been studied as a diagnostic and prognostic biomarker in disorders such as Alzheimer's disease (AD) [7, 8, 9], Creutzfeldt‐Jakob disease (CJD) [10, 11, 12, 13, 14] and Huntington's disease (HD) [15], as well as a prognostic biomarker in stroke and traumatic brain injury (TBI) [16, 17]. In a pilot study, cerebrospinal fluid (CSF) tau was elevated in patients with spinocerebellar ataxia type 2 (SCA2) and multiple system atrophy, cerebellar type (MSA‐c), compared to controls [18]. In SCA3, dysregulation of *MAPT* splicing with a decreased 4R/3R ratio has been observed [19].

Glial fibrillary acidic protein (GFAP) is the principal intermediate filament in mature astrocytes and has been investigated as a marker of astrocytic activation in AD, Parkinson's disease and amyotrophic lateral sclerosis [20]. Inflammation is known to be part of the neurodegenerative process in SCA3 [21], and astrocytes may play an important role, especially in early stages [22, 23].

Ubiquitin carboxy‐terminal hydrolase L1 (UCHL1) is one of the most abundant proteins in the brain [24] and, similarly to ataxin‐3, intervenes in ubiquitination pathways [25]. UCHL1 has been linked to pathological processes in AD and Parkinson's disease [24]. Together with GFAP, UCHL1 has been proposed as a biomarker in TBI [26, 27].

Neurofilament light‐chain (NfL) has emerged as an attractive biomarker in several neurological diseases [11, 28, 29, 30, 31]. In SCA3, NfL has been shown to be a promising biomarker when measured with a single‐plex assay in several cohorts [32, 33, 34], and with single‐plex and homebrew duplex assays in cohorts partially replicated in the present study [4, 35].

The aim of this study was to investigate whether plasma levels of tau, GFAP and UCHL1 differ in SCA3 carriers compared to controls, and to determine which variables influence the levels of such markers. Understanding these aspects could clarify the potential role of these molecules in future clinical trials. In addition, a secondary aim of this study was to replicate the results for NfL in an SCA3 cohort using a multiplex assay.

## METHODS

### Study participants

Our study comprised a main cohort and a replication cohort (Table S1). The main cohort consisted of 143 *ATXN3* mutation carriers and 172 unrelated healthy controls. The former group was divided into 23 pre‐ataxic carriers (Scale for the Assessment and Rating of Ataxia [SARA] score <3) and 120 ataxic patients (SARA score ≥3). Mutation carriers and 56 controls were recruited through the European Spinocerebellar ataxia type 3/Machado–Joseph disease Initiative (ESMI) study [36], between November 2016 and January 2019. Additional age‐matching control samples were obtained from two local repositories. ESMI participants underwent a standardized protocol including SARA [37], the Inventory of Non‐Ataxia Signs (INAS) [38], the Activities of Daily Living (ADL) questionnaire [39] and the Spinocerebellar Ataxia Functional Index (SCAFI) [40]. For mutation carriers who did not report an onset of gait ataxia, age of ataxia onset was predicted using a published formula for European populations [41]. A replication cohort was recruited via the Ataxia Biomarker Study Group from March 2018 to June 2019. This included healthy controls (*n* = 34) and pre‐ataxic (*n* = 4) and ataxic (*n* = 41) *ATXN3* mutation carriers. All centres received ethical approval from their local ethics committees. Written informed consent was obtained from all participants prior to enrolment.

### Sample collection

Blood samples were collected using ethylenediaminetetraacetic acid (EDTA) tubes and cell preparation tubes (BD Vacutainer CPT mononuclear cell preparation tube, sodium heparin), following a common protocol. CPT tubes were centrifuged at 1700*g* for 30 min at room temperature. Whole blood and plasma aliquots were then stored at −80°C. Participants’ *ATXN3* genotype was determined as previously reported [35]. All mutation carriers had been genetically diagnosed, and the CAG repeat length was available for 22 pre‐ataxic carriers and 105 ataxic patients in the main cohort. CSF samples were obtained via lumbar puncture for 11 *ATXN3* mutation carriers (three pre‐ataxic carriers and eight ataxic patients), following standard procedures. CSF samples were centrifuged at 1100*g* for 10 min, and aliquots were stored at −80°C.

### Biomarker quantification

Plasma samples were analysed using the Neurology 4‐Plex ‘A’ kit on the Simoa HD‐1 analyser (Quanterix, Billerica, MA, USA) at University College London and Mayo Clinic, following the manufacturer’s instructions. The Simoa platform is an ultrasensitive digital enzyme‐linked immunosorbent assay (ELISA) as previously described [42]. The Neurology 4‐Plex ‘A’ kit allows the simultaneous quantification of total tau (t‐tau), NfL, GFAP and UCHL1 [43]. For each sample, measurements were performed in duplicate, and average values with a coefficient of variation below 20% were considered. CSF t‐tau and phosphorylated tau^181^ (p‐tau^181^) were quantified with conventional ELISA using, respectively, the Innotest hTau Ag assay and the Innotest Phospho‐Tau(181P) kit (Fujirebio Europe N.V., Gent, Belgium).

### Statistical analysis

Data analysis was performed using Stata15.1 (StataCorp, Texas, USA). Quantitative variables are presented as mean (SD) or median (minimum, maximum). Categorical variables are presented as percentages. Differences in means were analysed with ANOVA (with Bonferroni correction for ad hoc comparisons), Wilcoxon's rank sum test or the *t* test. Analyte concentrations were transformed using the natural logarithm due to their right‐skewed distributions. Spearman's rho was used to examine correlations between CSF and plasma concentrations.

Effects of participant categories in biomarker concentrations were assessed with multiple linear regression, adjusting for confounders (age and sex) and first‐order interactions between participant categories and the confounders. Dependent variables were the log‐concentrations of the different analytes. Interaction terms were included if statistically significant (*p *< 0.05), whereas confounders were retained if they caused clinically significant changes in the main effect coefficients (more than 10%). Effects were reported as regression coefficients with their 95% confidence intervals (CIs) and *p* value. Classification performance of NfL was assessed through multiple logistic regression, with the logit(*y* = ataxic) as dependent variable, and the respective receiver operating characteristic (ROC) curve.

Multiple linear regression models were used to investigate the predictors of the biomarkers’ concentrations in mutation carriers. Maximum models were fitted using available variables (age, sex, disease duration, number of CAG repeats, SARA, INAS, ADL, SCAFI) and all the possible equations were examined. Model selection was based on Mallows’ *C*_p_ criterion, adjusted *R* ^2^ and the principle of parsimony. The proportion of explained variability was expressed using adjusted *R* ^2^ values. Model assumptions were evaluated for all reported models.

### Transgenic and wild‐type (WT) animals

Machado–Joseph disease transgenic mice (MJDTg; C57BL/6 background), expressing the N‐terminal‐truncated human ataxin‐3 with 69 glutamine repeats [44], were maintained as previously described [45]. MJDTg and WT (C57BL/6) mice were selected at 4–5 weeks (four MJDTg mice and four WT mice) and 8–9 weeks (three MJDTg mice and three WT mice). MJDTg mice show an early phenotype at the age of 4–5 weeks consisting of reduced body weight, worse performance in the rotarod and cerebellar atrophy. Whilst weak and diffuse accumulation of ataxin‐3 is observed at early time‐points, inclusion bodies appear around 6 weeks of age (P40), with increasing size and number thereafter [44]. The experiments were carried out in accordance with the European Community Council Directive (2010/63/EU) for the care and use of laboratory animals and previously approved by the Responsible Organization for the Animals Welfare of the Faculty of Medicine and Center for Neuroscience and Cell Biology of the University of Coimbra (ORBEA and FMUC/CNC, Coimbra, Portugal) and the national authority Direcção Geral da Agricultura e Veterinária (DGAV, Portugal).

### Cerebellar tissue collection and western blotting

Animals were given a ketamine/xylazine overdose (2 × 100 mg/kg ketamine + xylazine 10 mg/kg, intraperitoneal) and were transcardially perfused with phosphate buffered saline. Cerebella were dissected and stored at −80°C before use. Protein extracts and western blots were performed as previously described [45]. For these experiments, 15 µg of total protein extracts were run in the gel. A monoclonal anti‐tau (Tau46; 1:1000; Cell Signaling Technology) or mouse monoclonal anti‐β‐actin antibody (clone AC74; 1:5000; Sigma‐Aldrich), diluted in blocking solution (5% non‐fat milk in TBS‐T), was used. An alkaline phosphatase‐linked antibody specific to mouse immunoglobulin G (1:20,000, Amersham Biosciences, GE Healthcare, UK) was used as secondary antibody. GraphPad Prism v.8.0.1 was used to analyse animal data.

## RESULTS

### Plasma t‐tau concentrations are elevated in young pre‐ataxic and ataxic *ATXN3* mutation carriers compared to controls

**FIGURE 1:**
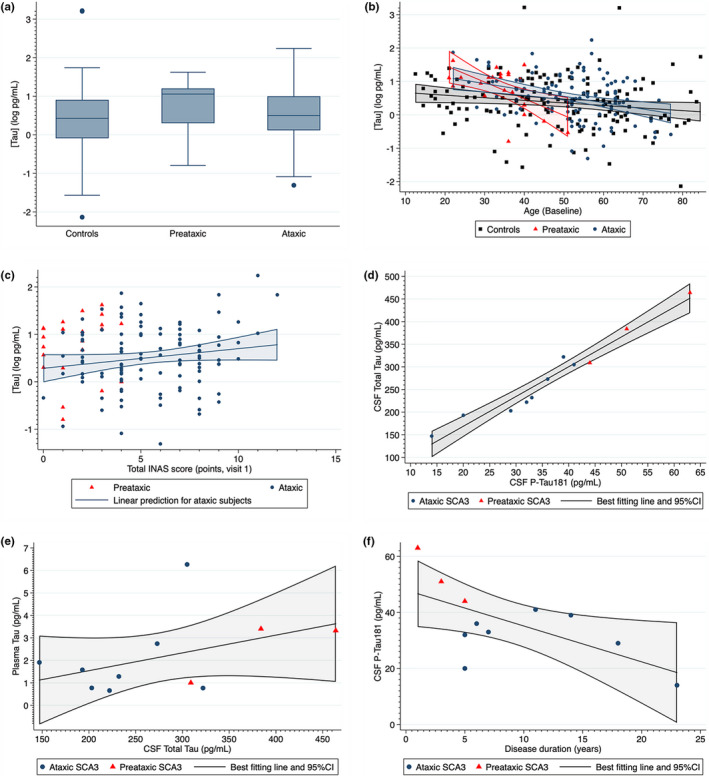
Total tau (t‐tau) in SCA3 (main cohort). (a) Plasma t‐tau concentrations in the control, pre‐ataxic and ataxic SCA3 groups. T‐tau values are expressed in the natural logarithmic scale. (b) Scatterplot showing the relationship between plasma t‐tau concentrations (in the natural logarithmic scale) and age for the different subject groups, with best fitting lines for controls (black squares), pre‐ataxic SCA3 (red triangles) and ataxic SCA3 (blue dots). The shaded areas represent the 95% CI of the best fitting lines. (c) Scatterplot showing the relationship between plasma t‐tau concentrations (in the natural logarithmic scale) and INAS count for *ATXN3* mutation carriers, with best fitting line and its 95% CI for the ataxic patients (blue dots). INAS was not investigated as a predictor in pre‐ataxic carriers (red triangles) due to its small range of variation (0–4) and its floor effect. (d) Scatterplot between CSF t‐tau concentrations (in pg/ml) and CSF p‐tau^181^ concentrations (in pg/ml), with best fitting line and its 95% CI for the pooled group of pre‐ataxic (red triangles) and ataxic (blue dots) SCA3 carriers. (e) Scatterplot between plasma t‐tau concentrations (in pg/ml) and CSF t‐tau concentrations (in pg/ml), with best fitting line and its 95% CI for the pooled group. Similar findings were obtained with p‐tau^181^ (data not shown). (f) Scatterplot between CSF p‐tau^181^ concentrations (in pg/ml) and disease duration (in years), with best fitting line and its 95% CI for the pooled group [Colour figure can be viewed at wileyonlinelibrary.com]

Both study cohorts are described in Table 1. Unadjusted plasma t‐tau concentrations were higher in pre‐ataxic carriers (0.77 log pg/ml [0.65]) compared to controls (0.37 log pg/ml [0.77]; *p *= 0.046), but they did not differ between ataxic SCA3 patients (0.51 log pg/ml [0.67]) and controls (*p *= 0.374) or between pre‐ataxic and ataxic *ATXN3* mutation carriers (*p *= 0.377) (Figure 1a). After adjustment for age and sex, an interaction between the groups and age was detected (Figure 1b and Table S2). Compared to controls, significantly higher t‐tau levels were found in pre‐ataxic carriers for 30 years of age (0.42 log pg/ml higher compared to controls; 95% CI 0.07, 0.77; *p *= 0.020) and for ataxic patients for 30 (0.42 log pg/ml; 95% CI 0.10, 0.73; *p *= 0.011) and 40 years of age (0.29 log pg/ml; 95% CI 0.07, 0.51; *p *= 0.010). In addition, it was observed that plasma t‐tau levels decreased with increasing age, with a steeper reduction in *ATXN3* mutation carriers.

**TABLE 1:** Participant characteristics for the different cohorts

	Main cohort					Replication cohort			
	Healthy controls	Pre‐ataxic SCA3	Ataxic SCA3	*p* value	CSF subcohort	Healthy controls	Pre‐ataxic SCA3	Ataxic SCA3	*p* value
Sample size (participants)	172	23	120		11	34	4	41	
Age (years)	48.5 (12.5, 85.5)	35.0 (21.0, 51.0)	52.0 (22.0, 77.0)	<0.001^a^	40.0 (24.0, 63.0)	54.7 (25.9, 79.2)	32.1 (30.7, 66.5)	52.6 (27.1, 75.2)	0.122^a^
Proportion of females (%)	52.6	56.5	57.5	0.703^b^	72.7	55.9	75.0	63.4	0.670^b^
No. of CAG repeats in the expanded *ATXN3* allele	N/A	69.0 (62.0, 73.0) (*n* = 22)	69.0 (56.0, 78.0) (*n* = 105)	0.562^c^	70.0 (61.0, 72.0) (*n* = 11)	N/A	65.0 (59.0, 69.0) (*n* = 4)	69.0 (51.0, 75.0) (*n* = 41)	0.156^c^
Age of onset (years)	N/A	37.7 (31.0, 57.4)	40.0 (14.0, 69.0)	0.604^c^	35.0 (19.0, 46.0)	N/A	48.1 (39.8, 68.8)	39.0 (18.0, 65.0)	0.187^c^
Disease duration (years)	N/A	−9.1 (−20.9, 5.0)	11.0 (−4.3, 39.0)	<0.001^c^	6.0 (1.0, 23.0)	N/A	−12.2 (−16.7, −2.3)	11.4 (3.6, 38.2)	0.001^c^
SARA score (points)	N/A	1.0 (0.0, 2.5)	13.5 (3.0, 37.0)	<0.001^c^	9.5 (1.0, 22.5)	N/A	0.0 (0.0, 0.0)	12.5 (5.0, 34.0)	0.001^c^
INAS count (points)	N/A	1.0 (0.0, 4.0)	5.5 (0.0, 12.0)	<0.001^c^	5.0 (3.0, 9.0)	N/A	N/A	N/A	N/A
ADL score (points)	N/A	0.0 (0.0, 4.0)	12.0 (0.0, 35.0)	<0.001^c^	9.0 (2.0, 22.0)	N/A	N/A	N/A	N/A
SCAFI score (*Z*‐score)	N/A	0.69 (−0.01, 2.42)	−0.30 (−2.67, 1.67)	<0.001^c^	0.07 (−1.75, 1.62)	N/A	N/A	N/A	N/A

Note

All the quantitative variables are expressed as median (minimum, maximum). Proportions are expressed as percentages.

Abbreviations: ADL, Activities of Daily Living; CSF, cerebrospinal fluid; INAS, Inventory of Non‐Ataxia Signs; N/A, not applicable; SARA, Scale for the Assessment and Rating of Ataxia; SCA3, spinocerebellar ataxia type 3; SCAFI, Spinocerebellar Ataxia Functional Index.

^a^ ANOVA test for the three participant categories (healthy controls, pre‐ataxic and ataxic mutation carriers).

^b^ Chi‐squared test.

c Wilcoxon's rank sum test between pre‐ataxic SCA3 and ataxic SCA3 carriers.

The different predictors for plasma t‐tau concentrations in pre‐ataxic and ataxic *ATXN3* mutation carriers were investigated separately. In pre‐ataxic carriers, age (−0.04 log pg/ml per 1‐year increase; 95% CI −0.07, −0.01; *p *= 0.008) and sex (0.51 log pg/ml increase in females compared to males; 95% CI 0.07, 0.95; *p *= 0.024) were the best predictors for plasma t‐tau, explaining 42.91% of its variability.

For ataxic SCA3 patients, age (−0.03 log pg/ml per 1‐year increase; 95% CI −0.04, −0.02; *p *< 0.001) and sex (0.36 log pg/ml increase in females compared to males; 95% CI 0.12, 0.60; *p *= 0.003) accounted only for 16.20% of the variability in plasma t‐tau. Adding the number of CAG repeats and the total INAS count increased the proportion of explained variability to 23.46%. INAS had a significant effect of 0.07 log pg/ml per 1‐point increase (95% CI 0.02, 0.12; *p *= 0.004) (Figure 1c). Therefore, patients with higher INAS scores (reflecting a more complex neurological phenotype) will tend to display higher plasma t‐tau concentrations. Although the coefficient for the number of CAG repeats was not significant (−0.04 log pg/ml per 1‐CAG increase; 95% CI −0.08, 0.01; *p *= 0.090), this variable increased the proportion of explained variability and was included in the best predictive model. No significant relationships were found with SARA or ADL and the inclusion of these variables did not produce better models.

To investigate if tau could be increased in early stages of SCA3, t‐tau and p‐tau^181^ levels were measured in our pilot CSF cohort. Median t‐tau levels in pre‐ataxic subjects (384 pg/ml [309, 464]) were higher than in ataxic patients (227 pg/ml [147, 322]; *p *= 0.025). CSF t‐tau concentrations in all the pre‐ataxic carriers were above 300 pg/ml, which has been reported as the cut‐off value for healthy controls between 21 and 50 years of age [46]. Likewise, CSF p‐tau^181^ levels in pre‐ataxic carriers (51 pg/ml [44, 63]) were greater than those in ataxic patients (32.5 pg/ml [14, 41]; *p *= 0.014). Greater CSF t‐tau concentrations were associated with higher CSF p‐tau^181^ levels in the pooled cohort (rho = 0.973; *p *< 0.001; Figure 1d). Plasma t‐tau concentrations were not associated with levels of either CSF t‐tau (rho = 0.318; *p* = 0.340; Figure 1e) or p‐tau^181^ (rho = 0.418; *p* = 0.201). CSF t‐tau and p‐tau^181^ were not associated with age (respectively, *p* = 0.537 and *p* = 0.450), but higher concentrations of p‐tau^181^ were associated with shorter disease duration (rho = −0.606 and *p* = 0.048 for p‐tau^181^, Figure 1f; rho = −0.551 and *p* = 0.079 for t‐tau, data not shown).

### Cerebellar t‐tau protein levels are increased in transgenic SCA3 mice in early stages of the disease

**FIGURE 2:**
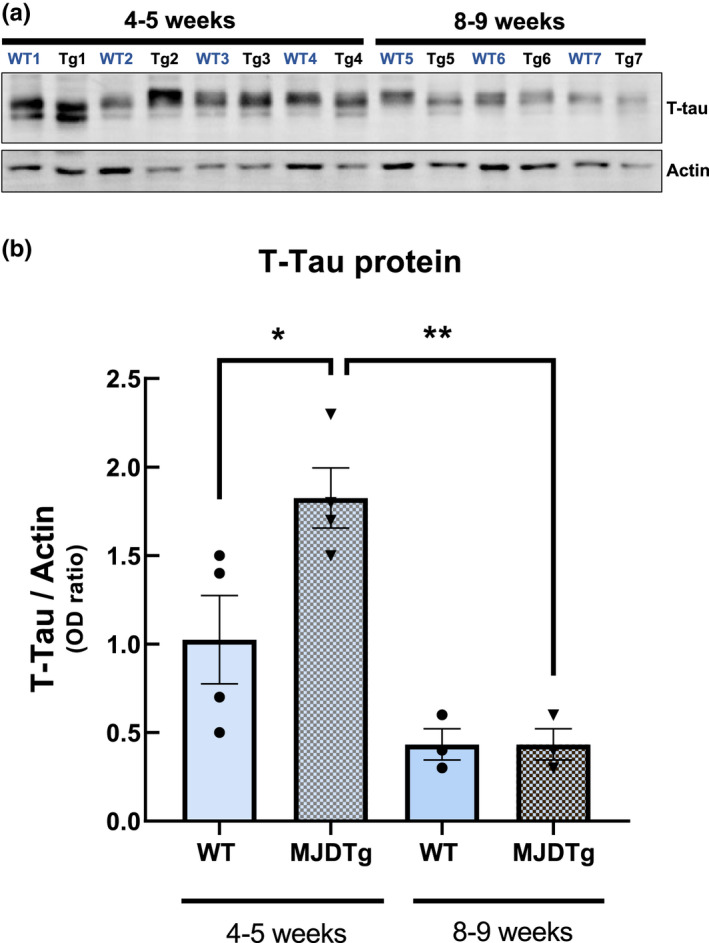
Total tau (t‐tau) protein levels are increased in the cerebella of SCA3 transgenic mice at an early symptomatic stage. Protein extracts from the cerebella of wild‐type (WT) and MJD transgenic mice (MJDTg) were analysed at 4–5 and 8–9 weeks of life by western blot (*n* = 3–4). (a) Membrane picture showing immunoreactivity against t‐tau and actin. (b) Optical densitometry (OD) analysis of t‐tau. Data were normalized to the housekeeping gene actin. Data are presented as mean ± SEM and normalized to 4–5‐weeks‐old WT mice. One‐way analysis of variance (ANOVA) followed by Sidak's post hoc test. *p = 0.033, **p = 0.001 [Colour figure can be viewed at wileyonlinelibrary.com]

Since higher plasma t‐tau concentrations were found in young pre‐ataxic and ataxic *ATXN3* mutation carriers compared to controls, and possibly increased CSF t‐tau and p‐tau^181^ levels in pre‐ataxic carriers compared to ataxic patients, it was assessed whether this pattern was reproducible in relevant neurological tissues from an animal model of SCA3 (MJDTg) [44]. The levels of t‐tau in cerebellar lysates from MJDTg mice were higher than the levels in lysates from WT animals at the age of 4–5 weeks (*p* = 0.033; Figures 2 and S1). However, cerebellar t‐tau levels were no different between the two groups at the age of 8–9 weeks. There was a reduction in cerebellar t‐tau levels in both groups over time, and the difference between 4–5 week and 8–9 week MJDTg mice was statistically significant (*p* = 0.001). Therefore, cerebellar t‐tau levels in this animal model mirrored our findings in humans, since they showed an increase in early symptomatic MJDTg mice compared with WT mice, and t‐tau levels decreased with advancing age.

### Plasma GFAP and UCHL1 did not show higher levels in mutation carriers compared to controls

When plasma GFAP concentrations were adjusted by age and sex, carriers showed significantly lower concentrations compared to controls: −0.45 log pg/ml for pre‐ataxic carriers (95% CI −0.71, −0.21; *p *< 0.001) and −0.20 log pg/ml for ataxic patients (95% CI −0.33, −0.06; *p *= 0.004). Age and sex explained 23.35% of the variability in GFAP, with minimal increment when considering the type of subject (27.25%). GFAP showed a steady rise with increasing age, similar for the three groups. In the replication cohort, GFAP did not differ between groups.

Unadjusted plasma UCHL1 concentrations were lower in pre‐ataxic *ATXN3* mutation carriers (2.55 log pg/ml [1.10]) compared to controls (3.03 log pg/ml [1.44]; *p *= 0.046), but levels in controls and ataxic participants were similar (2.65 log pg/ml [1.21]; *p *= 0.374). However, these results were calculated using a smaller subset of samples with a coefficient of variation of <20% (43.7% of the total).

### Plasma NfL concentrations are raised in *ATXN3* mutation carriers compared to controls, and this difference is most marked for younger subjects

**FIGURE 3:**
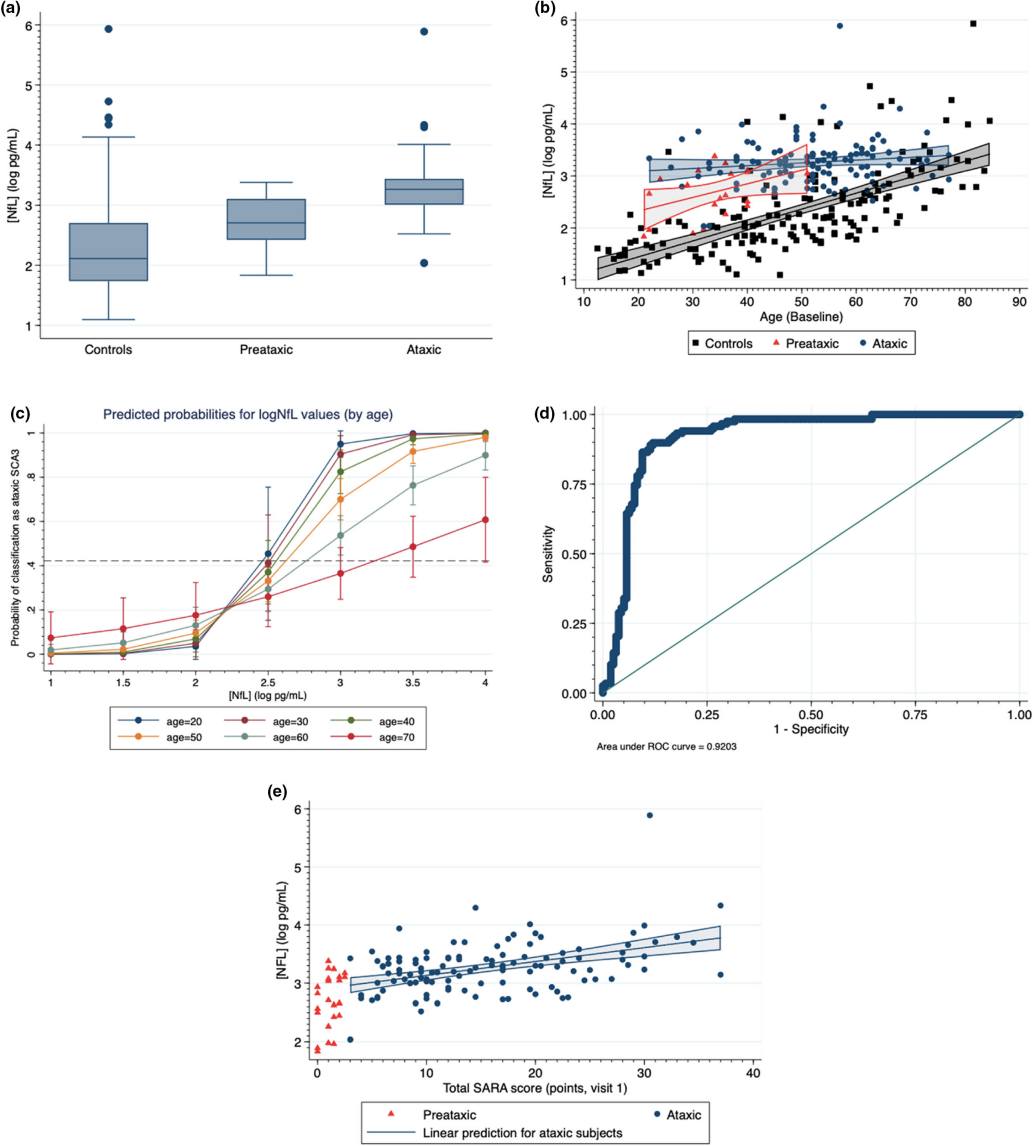
Neurofilament light‐chain (NfL) in SCA3 (main cohort). (a) Plasma NfL concentrations in the control, pre‐ataxic and ataxic SCA3 groups. NfL values are expressed in the natural logarithmic scale. (b) Scatterplot showing the relationship between plasma NfL concentrations (in the natural logarithmic scale) and age for the different participant groups, with best fitting line for each group (black squares, controls; red triangles, pre‐ataxic SCA3; blue dots, ataxic SCA3). The shaded areas represent the 95% CI of the best fitting lines. (c) Predicted probabilities of being classified as ataxic SCA3 (compared to controls), with logNfL as predictor, stratified for different age groups. The black dashed line indicates a probability of 0.42, the cut‐off threshold to be classified as ataxic SCA3. (d) ROC curve for the discrimination between ataxic SCA3 patients and controls, with NfL levels as predictor (adjusted by age and its interaction). The diagonal light blue line represents the null effect (AUC = 0.5). (e) Scatterplot showing the relationship between plasma NfL concentrations (in the natural logarithmic scale) and the SARA total score for *ATXN3* mutation carriers, with best fitting line and its 95% CI for ataxic patients (blue dots). SARA was not investigated as a predictor in the pre‐ataxic group (red triangles) due to its small range of variation (0–3) and its floor effect [Colour figure can be viewed at wileyonlinelibrary.com]

In the main cohort, unadjusted mean NfL levels in ataxic (3.26 log pg/ml [0.46]) and pre‐ataxic (2.70 log pg/ml [0.47]) *ATXN3* mutation carriers were higher than the value in controls (2.31 log pg/ml [0.83]; respectively, *p *< 0.001 and *p *= 0.032) (Figure 3a). Age and sex were included as confounders and an interaction between the participant categories and age was detected (Figure 3b). Therefore, differences in NfL between groups will vary depending on participants’ age, being greater in younger participants (Table S3).

The classification performance of NfL in differentiating ataxic SCA3 patients from controls was analysed through a multiple logistic regression model (adjusting by age and its interaction). In our model, a rise in one log‐unit in NfL increased the odds of being classified as ataxic, but such effect was attenuated with increasing age (coefficient for logNfL: 8.38–0.11 × (age), Figure 3c). The ROC curve for this model yielded an area under the curve (AUC) of 0.92 (95% CI 0.88, 0.95) (Figure 3d). When pre‐ataxic *ATXN3* mutation carriers and controls were compared, the model yielded an AUC of 0.89 (95% CI 0.83, 0.93). The inclusion of tau and its interaction with age did not produce models with improved AUC values.

In the replication cohort, unadjusted mean NfL reproduced the findings of the main cohort, with ataxic SCA3 (3.47 log pg/ml [0.36]) showing higher levels compared to controls (2.45 log pg/ml [0.53]; *p *< 0.001). Adjusted differences between ataxic SCA3 patients and controls were greater for younger subjects. The model to measure NfL classification performance yielded an AUC of 0.97 (95% CI 0.91, 1.00).

### Different variables predict plasma NfL in pre‐ataxic and ataxic *ATXN3* mutation carriers

The predictors of plasma NfL were investigated in ataxic and pre‐ataxic carriers separately. In ataxic SCA3 patients, both age (0.012 log pg/ml per 1‐year increase; 95% CI 0.002, 0.022; *p *= 0.022) and number of CAG repeats (0.034 log pg/ml per 1‐repeat increase; 95% CI 0.004, 0.064; *p *= 0.025) accounted only for 4.2% of the variability in NfL. In contrast, in pre‐ataxic carriers, the effects of age (0.051 log pg/ml per 1‐year increase; 95% CI 0.018, 0.084; *p *= 0.005) and number of CAG repeats (0.090 log pg/ml per 1‐repeat increase; 95% CI 0.018, 0.163; *p *= 0.018) were greater and explained 30.63% of NfL variation.

The effect of variables quantifying disease progression in ataxic patients was then investigated. Thus, it was found that the SARA score was associated with NfL (0.025 log pg/ml per 1‐point increase; 95% CI 0.015, 0.034; *p *< 0.001), yielding a percentage of explained NfL variability of 20.38% (Figure 3e). The effect of the INAS in NfL levels was non‐significant (*p *= 0.309). In the case of ADL, its effect (0.016 log pg/ml per 1‐point increase; 95% CI −0.001, 0.025; *p *= 0.082) was close to the threshold of significance and showed similar magnitude to the relationship found with SARA. The effect of the SCAFI was also significant (−0.173 log pg/ml per 1‐*Z*‐score increase; 95% CI −0.257, −0.089; *p *< 0.001), although this estimation was calculated with only 80.8% of the patients.

## DISCUSSION

The quantification of four different brain‐enriched proteins in a large SCA3 cohort composed of patients with diverse origins, ages and stages of the disease has been presented here. Our comprehensive control group allowed us to describe changes depending on age and define which subsets of patients were more likely to display significant changes.

Our results suggest that tau levels could be increased in *ATXN3* mutation carriers in early stages of the disease. First, in our main cohort, mutation carriers under 50 years of age showed higher plasma t‐tau concentrations compared to controls and their plasma levels decreased with increasing age. Secondly, CSF t‐tau and p‐tau^181^ concentrations could be elevated in pre‐ataxic *ATXN3* mutation carriers compared to ataxic patients. Finally, higher cerebellar t‐tau levels were found in early stages of the disease in MJDTg mice compared to young WT animals and MJDTg mice at a more advanced stage.

Increased CSF t‐tau has been found in various neurological conditions, such as AD [10, 11, 47], CJD [10, 11, 12, 13, 14], multiple system atrophy [18, 47], HD [15] and cases of encephalopathy/encephalitis [10, 11, 47]. Therefore, CSF t‐tau is considered a marker of neuronal damage elicited by different insults. In a recent report [48], CSF tau and p‐tau^181^ levels were similar between SCA3 patients and controls. However, in that study, the mean age of the two groups differed, pre‐ataxic carriers were not included and the differences were not age‐adjusted. Interestingly, our results might indicate that tau levels are elevated early in the disease course and decrease over time. In a previous study in AD, CSF t‐tau and p‐tau^181^ showed a reduction over time in a cohort of AD patients, which differed from the increase in the control and the mild cognitive impairment groups [49].

Plasma t‐tau has been shown to be elevated in AD patients compared to mild cognitive impairment and control participants and is associated with worse progression in different clinical and radiological variables in the AD group [9]. In CJD, plasma t‐tau showed a better classification performance than NfL comparing patients with CJD to patients with non‐prion rapidly progressive dementias, and higher plasma t‐tau levels were associated with a shorter survival in sporadic CJD patients for the subtype VV2 [12]. In this study, it was found that younger *ATXN3* mutation carriers showed higher t‐tau concentrations that decreased with increasing age. In our cohort, the INAS count was associated with plasma t‐tau levels. This might represent a relationship between plasma t‐tau and the complexity of the phenotype in SCA3. In addition, female carriers showed higher plasma t‐tau concentrations compared to males. In a previous natural history study, female *ATXN3* carriers were found to have a faster progression in the number of non‐ataxia signs compared to males [50]. This difference was only present for SCA3, and not for SCA1, SCA2 or SCA6. Therefore, the effect of the carrier's sex in plasma t‐tau could also be related to the complexity of the SCA3 phenotype.

Our results suggest an interesting link between tau and SCA3, and could represent a potential use of this protein as a marker of early disease stages. The pathogenic role of tau is being progressively unravelled for other polyglutamine disorders. In HD brains, different studies have found an increase in the 4R/3R isoform tau ratio [51, 52], with hyperphosphorylated tau nuclear rods in striatum and cortex (which partially co‐localize with mutant huntingtin) [51, 52]. In addition, the *MAPT* H2 haplotype was associated with a higher rate of cognitive decline in an HD cohort [52]. Several hypotheses could explain why tau levels are preferentially higher in early stages in SCA3. First, the neurodegenerative process might be more pronounced in early stages of the disease, with a subsequent stabilization when patients reach the fully symptomatic stage. A previous study found that several CSF markers of neuronal injury (including t‐tau) decreased over time in AD [49]. The authors considered whether this could be a consequence of the slowing of the neurodegenerative process or a result of the reduced number of neurons over time. In addition, they pointed out the discrepancies between imaging and biofluid markers, as the latter do not represent cumulative changes. Secondly, increased tau levels could reflect early pathophysiological changes in the neurodegenerative process. Tau has been shown to interact with vesicle‐associated proteins at presynaptic terminals and with proteins involved in mitochondrial bioenergetics [53]. Hypothetically, early synaptic and mitochondrial failure, as well as the cytoskeletal dysfunction in SCA3 [54, 55, 56], could elicit an increase in tau concentrations. In later stages, these phenomena would be sequentially overridden by axonal loss (mirrored by a progressive rise in NfL) and neuronal cell loss. Finally, there could be a progressive dysregulation of tau expression in the SCA3 disease course, as shown in a preliminary study [19]. Nonetheless, for its implementation as a biomarker in SCA3, further studies in tau kinetics in CSF and blood will be required, as well as longitudinal clinical studies in large patient cohorts.

In our cohort, plasma GFAP levels were not higher in *ATXN3* mutation carriers compared to controls. This could indicate that astrocytic activation is not a major component in SCA3 neurodegeneration, or that such activation is not translated into higher plasma levels. A previous report found increased plasma GFAP concentrations in SCA3 patients using a different methodology [57]. However, mean GFAP concentrations in our study were one to two orders of magnitude lower, which reflects the higher sensitivity of our method. Although there is evidence that GFAP transcription is increased with ageing [58], previous studies could not corroborate such age dependence of its peripheral levels. Our data confirm that plasma GFAP concentrations are age‐dependent and that age is an important predictor of GFAP. Although UCHL1 has shown some value in TBI [26, 27], the low reliability of our method with lower plasma values prevents valid conclusions being drawn regarding its role in SCA3.

The higher plasma NfL levels in *ATXN3* mutation carriers, measured using a multiplexed assay with other biomarkers, reproduced the findings of other studies in SCA3 [32, 33, 34], replicated the previously published results of NfL in a subset of samples from the present cohort measured with single‐plex and duplex assays [4, 35] and are in agreement with the results in other neurodegenerative disorders [11, 28, 29, 30, 31]. Interestingly, differences in plasma NfL between SCA3 carriers and controls were greater for younger participants. This is explained by the fact that NfL concentrations were elevated since early stages of the disease, whereas in control subjects NfL slowly rises with increasing age. If future treatments achieve a halt in the neurodegenerative process, younger patients might show greater absolute reductions in their plasma NfL concentrations compared to older patients. This would produce higher effect sizes in younger patients and therefore require a smaller sample size. The results in our main cohort were supported by the replication cohort, in which good classification performance of NfL in differentiating ataxic SCA3 from controls was also found.

From the set of variables analysed here, it can be concluded that age and number of CAG repeats accounted for almost a third of NfL variability in pre‐ataxic carriers and therefore that they may be important drivers of the neurodegenerative process in this group. Surprisingly, in ataxic patients, age and number of CAG were not strong determinants of NfL levels (4.2% of explained variability). Instead, variables that reflect disease status (e.g., SARA, SCAFI) were strongly associated and explained a higher proportion of variability. Therefore, clinical stage seems to be the main driver of NfL concentrations once the carrier has reached the ataxic stage. The association between plasma NfL concentrations and SARA has also been found in previous studies [33, 35]. However, the previously reported association between NfL and INAS was not found in this study [33], as the effect of the INAS score was not significant after adjustment by SARA. A good understanding of the determinants of NfL will be necessary for its use as progression biomarkers in future clinical trials. Our data suggest that, whilst NfL concentrations are likely to change with age in pre‐ataxic carriers, such change will not be as marked in ataxic patients, where NfL might be more stable over time, especially if the subject has a mild disease course. To confirm these hypotheses, longitudinal data in large cohorts and collection of other variables that inform on the rate of neurodegeneration (e.g., magnetic resonance imaging data) will be required. Since the predictors of NfL in preclinical and clinical phases might diverge, considering these two phases as separate groups might lead to better predictive models than the ones reported so far [32, 35].

Our study has some limitations. The cross‐sectional design prevented us from exploring changes in biomarker concentrations over time. Also, our pre‐ataxic group was reduced compared to the ataxic and control groups, and this poses difficulties in studying variables that influence biomarker concentrations. The CSF and animal data are limited, and these exploratory results should be replicated in larger groups. Another caveat is the poor association between CSF and plasma tau concentrations, which is in agreement with previous studies in other conditions [9, 59] and could indicate a different behaviour between CSF and plasma tau levels. Plasma t‐tau concentrations showed some overlap between *ATXN3* carriers and controls, and more sensitive methods or the quantification of specific isoforms might yield more informative results.

In conclusion, our results suggest that tau is elevated early in SCA3, and its levels decrease over time. This warrants further research to unravel the role of tau in SCA3 and its potential role as a marker of early stages. NfL has shown consistent results with other studies, with greater levels in *ATXN3* mutation carriers and associations with participants’ characteristics (age, number of CAG repeats) and clinical variables (SARA score, SCAFI). Therefore, NfL shows potential to be a good candidate as a biomarker for SCA3, which will need to be confirmed in longitudinal studies carried out in large cohorts before it can be implemented in clinical trials.

## AUTHOR CONTRIBUTIONS

**Hector Garcia‐Moreno:** Data curation (equal); formal analysis (lead); investigation (lead); project administration (equal); validation (equal); visualization (lead); writing—original draft (lead). **Mercedes Prudencio:** Data curation (equal); formal analysis (equal); investigation (lead); project administration (equal); validation (equal); visualization (equal); writing—original draft (lead). **Gilbert Thomas‐Black:** Data curation (equal); investigation (equal); writing—review and editing (equal). **Nita Solanky:** Investigation (equal); project administration (equal); writing—review and editing (equal). **Karen R. Jansen‐West:** Investigation (equal); validation (equal); writing—review and editing (equal). **Rana Hanna Al Shaikh:** Data curation (equal); investigation (equal); project administration (equal); writing—review and editing (equal). **Amanda Heslegrave:** Methodology (lead); resources (equal); supervision (equal); writing—review and editing (equal). **Henrik Zetterberg:** Methodology (lead); resources (equal); supervision (equal); writing—review and editing (equal). **Magda M. Santana:** Data curation (equal); formal analysis (equal); investigation (equal); project administration (equal); validation (equal); visualization (equal); writing—original draft (equal). **Luis Pereira de Almeida:** Conceptualization (equal); funding acquisition (equal); methodology (lead); resources (equal); supervision (equal); writing—review and editing (equal). **Ana Cristina Ferreira:** Investigation (equal); writing—review and editing (equal). **Cristina Januário:** Investigation (equal); project administration (equal); resources (equal); writing—review and editing (equal). **Jon Infante:** Investigation (equal); project administration (equal); resources (equal); writing—review and editing (equal). **Jennifer Faber:** Data curation (equal); investigation (equal); project administration (equal); writing—review and editing (equal). **Thomas Klockgether:** Funding acquisition (equal); resources (equal); supervision (equal); writing—review and editing (equal). **Kathrin Reetz:** Investigation (equal); project administration (equal); resources (equal); writing—review and editing (equal). **Mafalda Raposo:** Data curation (equal); investigation (equal); project administration (equal); writing—review and editing (equal). **Ana F. Ferreira:** Investigation (equal); writing—review and editing (equal). **Manuela Lima:** Funding acquisition (equal); resources (equal); supervision (equal); writing—review and editing (equal). **Ludger Schöls:** Funding acquisition (equal); investigation (equal); project administration (equal); resources (equal); supervision (equal); writing—review and editing (equal). **Matthis Synofzik**: Investigation (equal); writing—review and editing (equal). **Jeannette Hübener‐Schmid:** Investigation (equal); writing—review and editing (equal). **Andreas Puschmann:** Funding acquisition (equal); resources (equal); writing—review and editing (equal). **Sorina Gorcenco:** Data curation (equal); investigation (equal); project administration (equal); writing—review and editing (equal). **Zbigniew K. Wszolek:** Funding acquisition (equal); resources (equal); supervision (equal); writing—review and editing (equal). **Leonard Petrucelli:** Conceptualization (lead); funding acquisition (equal); resources (lead); supervision (lead); writing—review and editing (equal). **Paola Giunti:** Conceptualization (lead); funding acquisition (equal); resources (lead); supervision (lead); writing—review and editing (lead).

## CONFLICT OF INTERESTS

HZ is a Wallenberg Scholar supported by grants from the Swedish Research Council (#2018‐02532), the European Research Council (#681712), Swedish State Support for Clinical Research (#ALFGBG‐720931), the Alzheimer Drug Discovery Foundation (ADDF), USA (#201809‐2016862), and the UK Dementia Research Institute at UCL; has served at scientific advisory boards for Denali, Roche Diagnostics, Wave, Samumed, Siemens Healthineers, Pinteon Therapeutics and CogRx; has given lectures in symposia sponsored by Fujirebio, Alzecure and Biogen; and is a co‐founder of Brain Biomarker Solutions in Gothenburg AB (BBS), which is a part of the GU Ventures Incubator Program. LPdA has received grants from FCT Fundação para a Ciência e Tecnologia, grants from JPND Joint Programme for Neurodegenerative Diseases, grants from NAF National Ataxia Foundation, grants from pharma/biotech companies, outside the submitted work, has a patent WO/2020/144611 pending, a patent WO2018138371 pending, and a patent WO/2009/116884 issued. JI has received honoraria as speaker from Zambon and Exeltys. JF has received funding from the National Ataxia Foundation and as a fellow of the Hertie Academy for Clinical Neuroscience. TK has received research support from the Deutsche Forschungsgemeinschaft (DFG), the Bundesministerium für Bildung und Forschung (BMBF), the Bundesministerium für Gesundheit (BMG), the Robert Bosch Foundation, the European Union (EU) and the National Institutes of Health (NIH); has received consulting fees from Biohaven, Roche, UBC, Uniqure and Vico Therapeutics; has received speaker honoraria from Novartis and Bayer. KR has received honoraria for presentations or advisory boards from Lilly and Roche as well as clinical trial grants from Pfizer, Merck, Minoryx, Biogen and Roche; LS has received grants from EU—ERN‐RND registry (grant 947588); E‐rare/BMBF—Treat‐Ion (grant 01GM1907A); E‐rare/BMBF—TreatHSP (grant 01GM1905A); Innovationsfond—ZSE‐DUO (grant 01NVF17031); Innovationsfond—Translate NAMSE (grant 01NVF16024). AP has received personal fees from Elsevier Ltd, Oxford, U.K. ZKW has received grants from Abbvie Inc. (M15‐562 and M15‐563), Biogen Inc. (228PD201) and Biohaven Pharmaceuticals Inc. (BHV4157‐206 and BHV3241‐301). ZKW serves as PI of the Mayo Clinic American Parkinson Disease Association (APDA) Information and Referral Center. LP is a consultant for Expansion Therapeutics. PG has received grants and honoraria for advisory board from Vico Therapeutics, honoraria for advisory board from Triplet Therapeutics, grants and personal fees from Reata Pharmaceutical, grants from Wave.

## Data Availability

The data that support the findings of this study are available on reasonable request from the corresponding author. The data are not publicly available due to privacy or ethical restrictions.
