# Supplementary material for: Tau and neurofilament light‐chain as fluid biomarkers in spinocerebellar ataxia type 3
Source: Eur J Neurol. 2022 May 26;29(8):2439–52. doi: 10.1111/ene.15373 (PMC9543545; doi:10.1111/ene.15373)
Supplement: Supplementary file 1 [file ENE-29-2439-s001.docx]

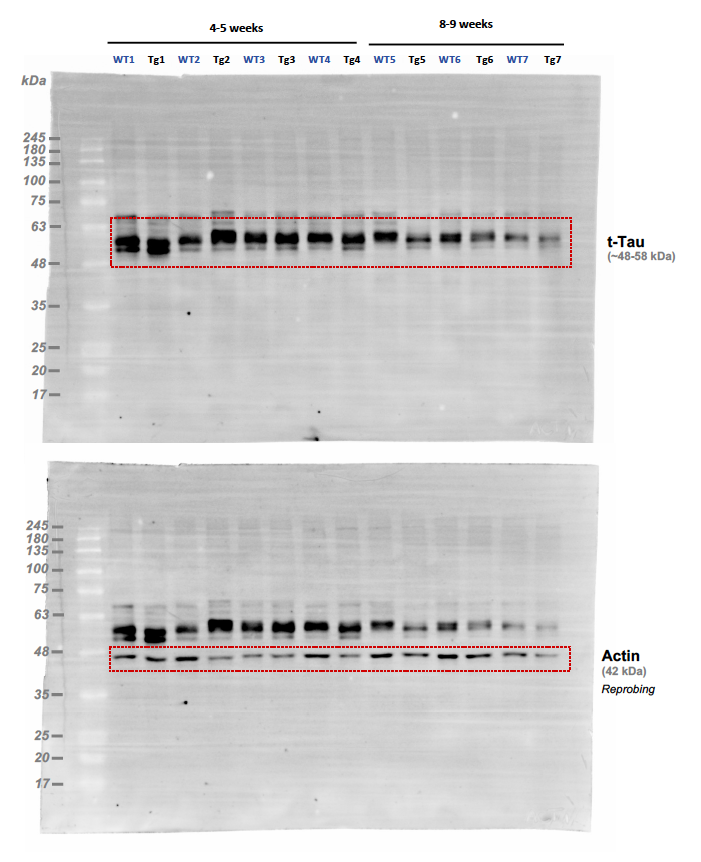


**Supplementary Figure 1. Uncropped western blot membrane for Figure 2.**

Red dashed lines represent the cropped sections shown in Figure 2 in the main manuscript
