# Supplementary material for: Tau and neurofilament light‐chain as fluid biomarkers in spinocerebellar ataxia type 3
Source: Eur J Neurol. 2022 May 26;29(8):2439–52. doi: 10.1111/ene.15373 (PMC9543545; doi:10.1111/ene.15373)
Supplement: Supplementary file 2 [file ENE-29-2439-s002.docx]

Supplementary Table 1. Composition of the main and replication cohorts.

| Site | SCA3 carriers (n) | Controls (n) |
| --- | --- | --- |
| Main cohort | | |
|  | | |
| *ESMI study* | | |
| University College London (United Kingdom) | 42 | 30 |
| University of Coimbra (Portugal) | 34 | 6 |
| University of Azores (Portugal) | 34 | 0 |
| University Hospital Bonn (Germany) | 19 | 10 |
| University of Tubingen (Germany) | 14 | 10 |
|  | | |
| *Local repositories* | | |
| UCL Bioresource, UCLH Biomedical Research Centre, NIHR (United Kingdom) | 0 | 106 |
| Centre for Adolescent Rheumatology versus Arthritis at UCL, UCLH and GOSH (United Kingdom) | 0 | 10 |
|  | | |
| TOTAL | 143 | 172 |
|  | | |
| Replication cohort | | |
|  | | |
| *Ataxia Biomarker Study Group* |  |  |
| Mayo Clinic Florida (USA) | 31 | 34 |
| Mayo Clinic Arizona (USA) | 1 | 0 |
| Lund University (Sweden) | 12 | 0 |
| University of Coimbra (Portugal) | 1 | 0 |
|  | | |
| TOTAL | 45 | 34 |

Supplementary Table 2. Differences in t-tau (log pg/mL) between groups, adjusted for age and sex in the main cohort.

|  | Preataxic SCA3 vs controls | | | Ataxic SCA3 vs controls | | |
| --- | --- | --- | --- | --- | --- | --- |
| Age of the participants | Differences between the groups | Ratio between the groups | p-value | Differences between the groups | Ratio between the groups | p-value |
| 30 | 0.42 (0.07, 0.77) | 2.31 (0.79, 3.84) | 0.020 | 0.42 (0.10, 0.73) | 2.30 (0.85, 3.76) | 0.011 |
| 40 | 0.05 (-0.32, 0.42) | 1.22 (-0.34, 2.77) | 0.780 | 0.29 (0.07, 0.51) | 2.19 (0.82, 3.56) | 0.010 |
| 50 | -0.31 (-0.98, 0.35) | -0.87 (-4.86, 3.12) | 0.353 | 0.16 (-0.01, 0.33) | 1.97 (0.41, 3.53) | 0.060 |
| 60 | N/A | N/A |  | 0.04 (-0.17, 0.24) | 1.40 (-1.21, 4.00) | 0.723 |

Differences in plasma t-tau concentrations between each *ATXN3* mutation carrier group (ataxic, n=108; preataxic, n=23) and controls (n=142) for different ages in males (reference category for sex). Selected values of age correspond to the range from the percentile 10 to the percentile 90 values for the distribution of age in the ataxic group. Effects for preataxic *ATXN3* mutation carriers were not calculated over 50 years of age, since the oldest preataxic carrier was 51 years old (N/A: not applicable). Differences are presented as regression coefficients of the effect of each group with the control group as reference category (95% CI), i.e., the differences in means between the two groups (95% CI of the difference). Ratios were calculated with the mean t-tau for the control group in males as reference. Their 95% CI were estimated with the delta method.

Supplementary Table 3. Differences in NfL (log pg/mL) between groups, adjusted for age and sex in the main cohort.

|  | Preataxic SCA3 vs controls | | | Ataxic SCA3 vs controls | | | Ataxic SCA3 vs preataxic SCA 3 | | |
| --- | --- | --- | --- | --- | --- | --- | --- | --- | --- |
| Age of the participants | Differences between groups | Ratio between groups | p-value | Differences between groups | Ratio between groups | p-value | Differences between groups | Ratio between groups | p-value |
| 30 | 0.83  (0.54, 1.11) | 1.49  (1.30, 1.67) | < 0.001 | 1.40  (1.15, 1.65) | 1.82  (1.64, 2.01) | < 0.001 | 0.57  (0.23, 0.91) | 1.23  (1.07, 1.38) | 0.001 |
| 40 | 0.80  (0.49, 1.10) | 1.40  (1.24, 1.56) | < 0.001 | 1.14  (0.97, 1.31) | 1.57  (1.46, 1.67) | < 0.001 | 0.34  (0.02, 0.67) | 1.12  (1.00, 1.25) | 0.036 |
| 50 | 0.77  (0.22, 1.31) | 1.33  (1.09, 1.57) | 0.006 | 0.88  (0.75, 1.02) | 1.38  (1.31, 1.45) | < 0.001 | 0.12  (-0.43, 0.67) | 1.04  (0.85, 1.22) | 0.675 |
| 60 | N/A | N/A |  | 0.63  (0.47, 0.78) | 1.24  (1.17, 1.31) | < 0.001 | N/A | N/A |  |
| 70 | N/A | N/A |  | 0.37  (0.14, 0.60) | 1.13  (1.04, 1.20) | 0.002 | N/A | N/A |  |

Differences in plasma NfL between each *ATXN3* mutation carrier group (ataxic, n=118; preataxic, n=23) and controls (n=157) and between both *ATXN3* mutation carrier groups for different ages in males (reference category for sex). Selected values of age correspond to the range from the percentile 10 to the percentile 90 values for the distribution of age in the ataxic group. Effects for preataxic *ATXN3* mutation carriers were not calculated over 50 years of age, since the oldest preataxic carrier was 51 years old (N/A: not applicable). Differences are presented as regression coefficients of the effect of each group compared to a reference group (controls or preataxic *ATXN3* mutation carriers) with their 95% CI, i.e., the differences in means between the two groups (with their 95% CI of the difference). Ratios were calculated with the mean NfL for the control or the preataxic group in males as reference. Their 95% CI were estimated with the delta method.

Supplementary Table 4. Co-investigators of the European Spinocerebellar ataxia type 3/Machado-Joseph disease Initiative (ESMI) consortium and the Ataxia Biomarker Study Group.

| Name | Affiliation |
| --- | --- |
| Bart P. van de Warrenburg | Radboud university medical centre, Donders Institute for Brain, Cognition and Behaviour, Department of Neurology, Nijmegen, The Netherlands |
| Judith van Gaalen | Radboud university medical centre, Donders Institute for Brain, Cognition and Behaviour, Department of Neurology, Nijmegen, The Netherlands |
| Jeroen J. de Vries | Department of Neurology, University of Groningen, University Medical Center Groningen, The Netherlands |
| Janna Krahe | Department of Neurology, RWTH Aachen University, Aachen, Germany |
| Heike Jacobi | Department of Neurology, University Hospital of Heidelberg, Heidelberg, Germany. |
| James Polke | Department of Neurogenetics, National Hospital for Neurology and Neurosurgery, University College London Hospitals NHS Foundation Trust, London, UK. |
| Robyn Labrum | Department of Neurogenetics, National Hospital for Neurology and Neurosurgery, University College London Hospitals NHS Foundation Trust, London, UK. |
| Yuping Song, MS | Department of Neuroscience, Mayo Clinic, Jacksonville, FL, USA |
| Judith A. Dunmore, MS | Department of Neuroscience, Mayo Clinic, Jacksonville, FL, USA |
| Björn Oskarsson, MD | Department of Neurology, Mayo Clinic, Jacksonville, FL, USA |
| Katharine A. Nicholson | Sean M. Healey and AMG Center for ALS, Massachusetts General Hospital (MGH), Boston, MA, USA |
| Nathan P. Staff, MD, PhD | Department of Neurology, Mayo Clinic, Rochester, MN, USA |
| Christin Karremo | Lund University, Skåne University Hospital, Department for Clinical Sciences Lund, Neurology, Lund, Sweden |
| João Lemos | Coimbra University Hospital Centre, Coimbra University, Coimbra, Portugal |
| Mark S. LeDoux, MD, PhD | University of Memphis and Veracity Neuroscience LLC, Memphis, TN, USA. |
| Joseph H. Friedman, MD | Department of Neurology, Warren Alpert Medical School of Brown University, Providence, RI, USA. |
| John D. Fryer | Neuroscience Graduate Program, Mayo Clinic Graduate School of Biomedical Sciences, Jacksonville, FL, USA. Department of Neuroscience, Mayo Clinic, Scottsdale, AZ, USA. |
| Christin Karremo | Lund University, Skåne University Hospital, Department of Clinical Sciences Lund, Neurology, Lund, Sweden |
| Inês Gomes | Coimbra University Hospital Centre, Coimbra University, Coimbra, Portugal |
| John N. Caviness | Department of Neurology, Mayo Clinic, Scottsdale, AZ, USA |
| Mark R. Pittelkow | Department of Dermatology, Mayo Clinic, Scottsdale, AZ, USA |
| Ronald F. Pfeiffer | Department of Neurology, Oregon Health & Science University, Portland, OR, USA. |
| Venka Veerappan | Department of Neurology, Oregon Health & Science University, Portland, OR, USA. |
| Eric R. Eggenberger | Department of Neurology, Mayo Clinic, Jacksonville, FL, USA |
| William D. Freeman, MD | Department of Neurology, Mayo Clinic, Jacksonville, FL, USA |
| Josephine F. Huang, MD | Department of Neurology, Mayo Clinic, Jacksonville, FL, USA |
| Ryan J. Uitti | Department of Neurology, Mayo Clinic, Jacksonville, FL, USA |
| Philip W. Tipton | Department of Neurology, Mayo Clinic, Jacksonville, FL, USA |
| Jay A. van Gerpen | University of Alabama at Birmingham, Birmingham, AL, USA. |
